# Doxycycline protects against ROS-induced mitochondrial fragmentation and ISO-induced heart failure

**DOI:** 10.1371/journal.pone.0175195

**Published:** 2017-04-06

**Authors:** Adam Riba, Laszlo Deres, Krisztian Eros, Aliz Szabo, Klara Magyar, Balazs Sumegi, Kalman Toth, Robert Halmosi, Eszter Szabados

**Affiliations:** 1 1st Department of Medicine, University of Pecs, Pécs, Hungary; 2 Szentagothai ResearchCenter, University of Pecs, Pécs, Hungary; 3 Department of Biochemistry and Medical Chemistry, University of Pecs, Pécs, Hungary; 4 MTA-PTE Nuclear-Mitochondrial Interactions Research Group, Pécs, Hungary; Azienda Ospedaliera Universitaria di Perugia, ITALY

## Abstract

In addition to their anti-bacterial action, tetracyclines also have complex biological effects, including the modification of mitochondrial protein synthesis, metabolism and gene-expression. Long-term clinical studies have been performed using tetracyclines, without significant side effects. Previous studies demonstrated that doxycycline (DOX), a major tetracyclin antibiotic, exerted a protective effect in animal models of heart failure; however, its exact molecular mechanism is still unknown. Here, we provide the first evidence that DOX reduces oxidative stress—induced mitochondrial fragmentation and depolarization in H9c2 cardiomyocytes and beneficially alters the expression of Mfn-2, OPA-1 and Drp-1 –the main regulators of mitochondrial fusion and fission—in our isoproterenol (ISO)–induced heart failure model, ultimately decreasing the severity of heart failure. In mitochondria, oxidative stress causes a shift toward fission which leads to mitochondrial fragmentation and cell death. Protecting mitochondria from oxidative stress, and the regulation of mitochondrial dynamics by drugs that shift the balance toward fusion, could be a novel therapeutic approach for heart failure. On the basis of our findings, we raise the possibility that DOX could be a novel therapeutic agent in the future treatment of heart failure.

## Introduction

Despite the development of many new treatments over the last 30 years, heart failure (HF) remains a major health problem in the western world. Drugs used in the antagonism of the neurohumoral system markedly reduce the mortality associated with heart failure, but further inhibition of this neurohormonal-cytokine axis have been unsuccessful [1, 2]. Therefore, targeting new therapeutic approaches is crucial to further reduce the morbidity and mortality associated with heart failure. Increasing evidence suggests that reactive oxygen species (ROS) play a major pathological role in the development and progression of heart failure [3]. Mitochondria are primarily responsible for ATP synthesis, but under pathophysiological conditions they become the main sources of endogenous ROS production [4]. The chronic exposure to ROS leads to further deterioration of heart muscle resulting in cardiac remodeling, apoptosis, necrosis, and fibrosis [5].

A balance in mitochondrial fusion and fission is essential in the maintenance of mitochondrial networks and critical for cardiac metabolism and heart function [6]. Oxidative conditions deteriorate this subtle balance [7]. Mitochondrial fusion and fission are regulated by dynamin-like guanosine triphosphatases (GTPases) such as Mfn-2, OPA-1 and Drp-1 [8, 9], which are markedly induced by oxidative stress [10]. Mitofusin 1 and 2 (Mfn-1 and Mfn-2) both induce outer mitochondrial membrane fusion and fission, whereas OPA-1 is required for inner mitochondrial membrane fusion [11]. Fission is also executed by dynamin-related protein 1 (Drp-1), a cytosolic protein that is recruited to the mitochondrial surface by phosphorylation in response to oxidative stress. It has also been well established that mitochondrial fragmentation occurs in heart failure [12–14].

Many studies have previously examined drugs targeting mitochondrial energy metabolism and ROS production, but most of these have failed to alleviate cardiac damage in clinical investigations, demonstrating the complexity of the processes occurring in the mitochondria [15]. Here we examined doxycycline (DOX), a tetracycline antibiotic which is a well-known antimicrobial [16] agent with minimal side-effects, even after long-term administration [17–19], as a possible new therapeutic agent for heart failure. DOX has a well established inhibitory effect on matrix metalloproteinases (MMP-2 and 9) [20, 21], which is believed to be the basis of its major protective effect in the acute phase of myocardial infarction and in the early phase of myocardial remodeling [22–24]. The aim of the present study was to further examine the protective effect of DOX in a postinfarction heart failure model and on a rat cardiomyocyte cell line.

## Methods

### Cell viability assay

The sulforhodamine B (SRB) assay is used for measuring drug-induced cytotoxicity and cell proliferation density, based on the measurement of cellular protein content. SRB assay was performed to determine the protective effect of DOX against H_2_O_2_ induced cardiomyocyte death. H9c2 rat heart myoblast cells were obtained from the European Collection of Cell Cultures (ECACC) and maintained in Dulbecco’s modified Eagle’s medium (DMEM) supplemented with 10% fetal bovine serum (FBS, Invitrogen, Carlsbad, CA, USA), 4 mM glutamine, 100 IU/ml penicillin and 100 ug/ml streptomycin. The effect of H_2_O_2_ and doxycycline on H9c2 cardiomyocytes was investigated using the Sulforhodamine B (SRB) assay, performed according to the method of Papazisis and colleagues [25], with modifications. H9c2 cells were cultured in 96-well microtiter plates in a humidified 95% O_2_/5% CO_2_ atmosphere at 37°C. Cells were treated for 2 hours with different concentrations of H_2_O_2_ (0.2 mM, 0.3 mM, 0.4 mM, 0.5 mM). Fifty percent of cell degradation was detected at the concentration of 0.3 mM H_2_O_2_. Increasing concentrations of doxycycline (50 nM, 100 nM, 300 nM, 1 μM, 10 μM) were applied to the cells in the subsequent study. Culture medium was aspirated prior to fixation of the cells by addition of 200 μl cold 10% trichloroacetic acid. After a 20 min incubation at 4°C, cells were washed 5 times with deionized water. Microplates were then left to dry at room temperature for at least 24 h. The cells were then stained with 200 μl 0.1% SRB dissolved in 1% acetic acid for at least 20 min at room temperature and subsequently washed 4 times with 1% acetic acid to remove unbound stain. The plates were left to dry at room temperature. Bound SRB was solubilized with 200 μl 10 mM unbuffered Tris-base solution and plates were left on a plate shaker for at least 10 min. Absorbance was read with a GloMax Multi Detection System (Promega, USA) at 560 nm subtracting the background measurement at 620 nm. The test optical density (OD) value was defined as the absorbance of each individual well, minus the blank value (‘blank’ is the mean optical density of the background control wells). All experiments were performed in triplicate [26, 27].

### Detection of mitochondrial fragmentation with fluorescent microscopy

The H9c2 rat heart myoblast cell line was obtained from the European Collection of Cell Cultures (Salisbury, UK). The cell line was maintained in a humidified 5% CO2 atmosphere at 37°C. H9c2 cells were cultured in Dulbecco's modified Eagle's medium (DMEM, PAA Laboratories, Cölbe, Germany) containing 10% fetal bovine serum (FBS) and antibiotic solution (1% penicillin and streptomycin mixture; Gibco/Invitrogen, Carlsbad, CA). Cells were passaged at 3 day intervals. H9c2 cells were seeded at a starting density of 1x105 cells/well in 6 well plates on glass coverslips and cultured at least overnight before the experiment. The next day, cells were washed twice in PBS, added fresh medium (containing 10% FBS) and then treated with H2O2 with or without doxycycline. Group of cells: Control: cells without any treatment; DOX: 5 μM doxycycline for 5 hours; H2O2: 400 μM H2O2 for 5 hours; H2O2+DOX: 400 μM H2O2 and 5 μM doxycycline for 5 hours. After subjecting cells to the appropriate treatment, coverslips were rinsed twice in modified Krebs-Henseleit solution. To visualize the mitochondria, 50 nM of MitoTracker Red (Molecular Probes) was added and incubated for 15 minutes. The cells were then washed twice in modified Krebs-Henseleit solution (containing 10% FBS) and placed upside down on the top of a small chamber formed by a microscope slide and a press-to-seal silicone isolator. The chamber was filled with modified Krebs-Henseleit solution containing 4.5 g/L glucose. The cells were visualized using a Nikon Eclipse Ti-U fluorescent microscope equipped with a Spot RT3 camera using a 60x objective and epifluorescent illumination. The investigator collecting data was blinded to treatment groups. Representative merged images of three independent experiments are presented.

### Mitochondrial membrane potential measurement with JC-1 assay for fluorescent microscopy

The mitochondrial membrane potential (ΔΨm) was measured using the mitochondrial membrane potential—specific fluorescent probe, JC-1 (Molecular Probes). H9c2 cells were seeded on glass coverslips and cultured at least overnight before the experiment. After treatment, cells were washed twice in ice-cold PBS and then incubated for 5 min at 37°C in modified Krebs-Henseleit solution (118mM NaCl, 5mM KCl, 1.2 mM KH_2_PO_4_, 25 mM NaHCO_3_, 5mM glucose, 1.2 mM MgSO_4_x7H_2_O) containing 0.1 μM JC-1. When excited at 488 nm, the dye emits green fluorescence (530 nm) at low ΔΨm and red (590 nm) at high ΔΨm. Following incubation, the cells were washed once with Krebs-Henseleit solution and then imaged with a Nikon Eclipse Ti-U fluorescent microscope equipped with a Spot RT3 camera using a 60x objective and epifluorescent illumination. The investigator collecting data was blinded to treatment groups. All experiments were repeated in triplicate.

### Animals

Male 16 week old Wistar rats (410–480 g) were used for the experiments. Animals received care according to the Guide for the Care and Use of Laboratory Animals published by the US National Institute of Health (NIH Publication No. 85–23, revised 1996) and the experiment was approved by the Animal Research Review Committee of the University of Pecs, Medical School (Permit number: BA02/2000-2/2010). The animals were housed under standardized conditions, 12 h dark—light cycle in solid bottomed polypropylene cages, and received commercial rat chew ad libitum. Water consumption of rats was measured for two weeks before the beginning of the experiment. The mean volume was 75ml/rat/day. Doxycycline or clear water was administered as drink ad libitum for 8 weeks. We set the dosage of doxycycline to 5 mg/b.w.(kg)/day [22, 28].

### Experimental protocol

To induce postinfarction myocardial remodeling, the rats were treated twice on two consecutive days with 80 mg/kg ISO (Sigma-Aldrich) or vehicle subcutaneously as previously described [29]. The animals were divided into the following four groups and were followed for 8 weeks: control group (C, n = 5), received clear water without any treatment; ISO group (ISO, n = 7) received two subcutaneous injections of ISO at a dosage of 80 mg/b.w.(kg) and clear water afterwards; ISO+doxycycline group (ISO+DOX, n = 7), received 5 mg/b.w.(kg)/day doxycycline after ISO treatment; doxycycline group (DOX, n = 6), received 5 mg/b.w.(kg)/day doxycycline without ISO treatment. In the ISO—treated groups, 24 hours after the second injection the surviving animals were randomly assigned to receive either doxycycline (5 mg/b.w(kg)/day or clear water. At the end of the 8 week long treatment period, body weights were measured, animals were sacrificed and the hearts were removed. The atria and great vessels were trimmed from the ventricles and the weight of the ventricles was measured and normalized to body mass and tibia length. The ventricles were fixed in 10% formalin for histology or freeze clamped for Western blot analysis.

### Determination of plasma B-type natriuretic peptide level

Blood samples were collected into Vacutainer tubes containing EDTA and aprotinin (0.6 IU/ml) and centrifuged at 1600 g for 15 minutes at 4°C to obtain plasma, which was collected and kept at -70°C. Plasma B-type natriuretic peptide-45 levels (BNP-45) were determined by enzyme immunoassay (BNP-45, Rat EIA Kit, Phoenix Pharmaceuticals Inc., CA, USA) [28].

### Non-invasive evaluation of cardiac function

The investigators were blinded to the treatment protocol. Under baseline conditions, all animals were examined by means of echocardiography to exclude rats with any pre-existing heart abnormalities. Transthoracic two-dimensional echocardiography was performed under inhalation anesthesia at the beginning of the experiment and on final day, prior to sacrificing the animals. The rats were lightly anesthetized with a mixture of 1.5% isoflurane and 98.5% oxygen. The chest of each animal was shaved, acoustic coupling gel was applied, and a warming pad was used to maintain normothermia. The animals were imaged in the left lateral position. Cardiac diameters and function were measured parasternal from the short- and long-axis views at the midpapillary level by using a VEVO 770 high-resolution ultrasound imaging system (VisualSonics, Toronto, Canada), which was equipped with a 37.5-MHz transducer. Left ventricular (LV) systolic function (ejection fraction-EF), LV end-diastolic volume (LVEDV), LV end-systolic volume (LVESV), as well as the thickness of the septum and posterior wall (PW) were determined. EF (%) was calculated as 100x[(LVEDV—LVESV)/ LVEDV] [28].

### Histology

After fixation in formalin, the ventricles were sliced and embedded in paraffin. Sections (5 μm thick) were cut serially from base to apex. Sections were stained with Masson’s trichrome stain to detect interstitial fibrosis, and quantified by the NIH ImageJ image processing program via its color deconvolution plugin [28]. All histological samples were examined by an investigator in a blinded fashion.

### Nitrotyrosine immunohistochemical staining

We performed immunohistochemical staining for nitrotyrosine (Milipore #06–274, rabbit polyclonal 1:100), a marker of nitrooxidative stress, using a previously described method [30]. Extensively stained area was also quantified using the NIH ImageJ image processing program via its color deconvolution plugin.

### Western blot analysis

Fifty milligrams of heart samples were homogenized in ice-cold Tris buffer (50 mmol/l, pH 8.0) containing 50 mM sodium vanadate and protease inhibitor cocktail (Sigma-Aldrich Co., Budapest, Hungary) and harvested in 2× concentrated SDS-polyacrylamide gel electrophoresis sample buffer. Proteins were separated on a 12% SDS-polyacrylamide gel and transferred to nitrocellulose membranes. After blocking (2 h with 3% nonfat milk in Tris-buffered saline), membranes were probed overnight at 4°C with antibodies recognizing the following antigens: mitofusin-2 (Mfn-2; 80 kDa; 1:1000; Cell Signaling #9482), optic atrophy protein-1 (OPA-1; 100–110 kDa, 1:1000; Abcam ab42364), Drp-1(Cell Signaling, #8570S) and phospho-specific Drp-1 Ser616 (Cell Signaling, #3455S) (95 kDa; 1:1000). GAPDH was used as loading control (Sigma, #105M4803, 1:1000) Membranes were washed six times for 5 min in Tris-buffered saline (pH 7.5) containing 0.2% Tween (TBST) before addition of goat anti-rabbit horseradish peroxidase-conjugated secondary antibody (1:3000 dilution; Bio-Rad, Budapest, Hungary). Membranes were washed six times for 5 min in TBST and the antibody—antigen complexes were visualized by means of enhanced chemiluminescence. The results of Western blots were quantified using the NIH ImageJ program.

### Statistical analysis

Statistical analysis was performed by analysis of variance and all of the data were expressed as the mean ± SEM. The homogeneity of the groups was tested by F-test (Levene’s test). There were no significant differences among the groups. Comparisons among groups were performed using one-way ANOVA followed by Bonferroni correction in SPSS for Windows, version 21.0. All data are expressed as mean ± S.E.M. A value of p < 0.05 was considered statistically significant.

## Results

### Protective effect of doxycycline against the free radical–induced injury of cardiomyocytes

**Fig 1:**
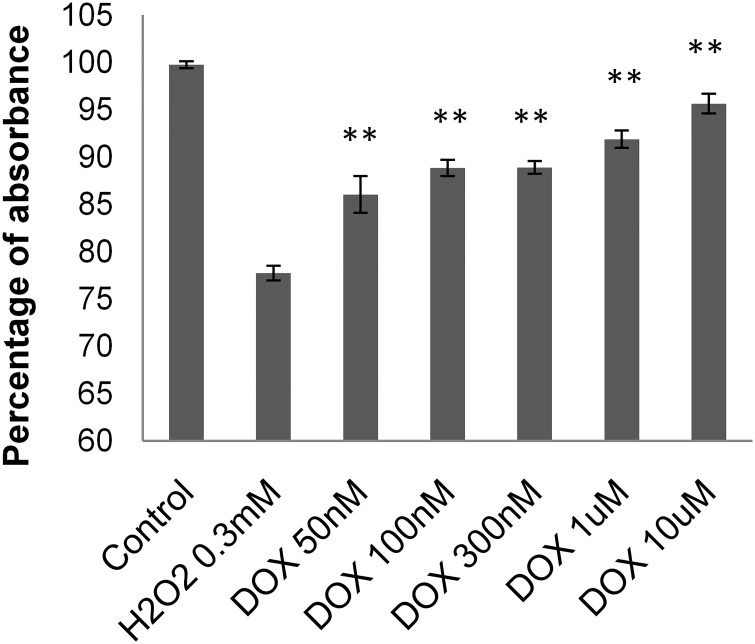
DOX protects against the H_2_O_2_–induced cell death. A wide range of doxycycline concentrations (50 nM, 100 nM, 300 nM, 1 μM, 10 μM) were applied to H_2_O_2_ –stressed (0.3 mM, 3h) H9c2 cells. All concentrations of DOX significantly improved the cell viability (**P < 0.01).

H9c2 cardiomyocytes were exposed to different concentrations of H_2_O_2_ for 24 hours. Fifty percent of myocyte cell death was detected at a concentration of 0.3 mM H_2_O_2_. Increasing concentrations of DOX (50 nM, 100 nM, 300 nM, 1 μM, 10μM) were able to significantly improve cell survival (P < 0.01; Fig 1).

### Doxycycline attenuates oxidative stress–induced mitochondrial fragmentation

**Fig 2:**
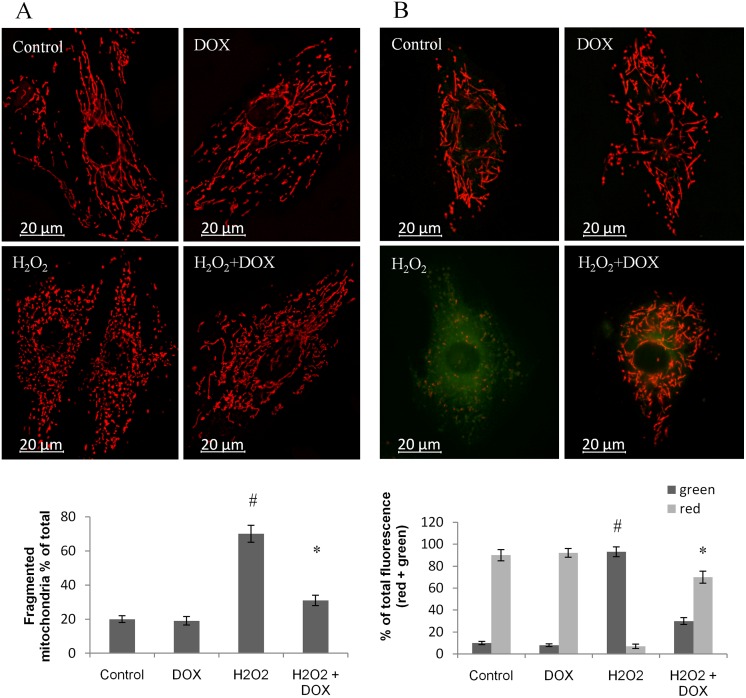
Mitochondrial depolarization and fragmentation in H9c2 cardiomyocytes. **(A)** Mitochondrial fragmentation was induced in H9c2 cells after incubation with 400 μM H_2_O_2_ for 5 hours, at which time the mitochondrial filaments disappeared and fragmented mitochondria with lengths shorter than 2 μm were observed instead. Doxycycline reduced or completely prevented ROS—induced mitochondrial fragmentation at the concentration of 5 μM. **(B)** DOX protected H9c2 cells from apoptosis by preventing the depolarization of the mitochondrial membrane. Green and red fluorescence images of the same field were acquired using a fluorescent microscope equipped with a digital camera. The images were merged to demonstrate depolarization of Δψ in vivo, indicated by loss of the red component of the merged image. Some red fragments can also be seen, representing the fragmented mitochondria. Representative merged images of three independent experiments are presented (#P < 0.05, C vs. H_2_O_2_ and *P < 0.05, H_2_O_2_ vs. DOX+H_2_O_2_).

Hydrogen peroxide induces mitochondrial fission resulting in fragmented mitochondria within 4 to 6 hours. This can be detected with MitoTracker dyes, or mitochondria—directed fluorescent proteins. The effect of doxycycline on hydrogen peroxide—induced mitochondrial fragmentation was analyzed by labeling the mitochondria with mitochondria—directed Advanced Red Fluorescent Protein (mARFP). Mitochondrial fragmentation was induced in H9c2 cells after incubation with 400 μM H_2_O_2_ for 5 hours, at which time the mitochondrial filaments disappeared and fragmented mitochondria with lengths shorter than 2 μm were observed instead. Doxycycline reduced ROS—induced mitochondrial fragmentation at a concentration of 5 μM (P < 0.05, C vs. H_2_O_2_ and P < 0.05, H_2_O_2_ vs. DOX+ H_2_O_2;_ Fig 2A).

### Doxycycline hyperpolarizes the mitochondrial membrane of H9c2 cardiomyocytes

We studied the mitochondrial membrane potential of control, DOX (5 μM), H_2_O_2_ (400 μM), and H_2_O_2_+DOX—treated cells. After a 5 hour long treatment, cells were loaded with the voltage—sensitive fluorescent mitochondrial dye—JC1 –then red and green fluorescence was determined by fluorescent microscopy. DOX effectively prevented mitochondrial membrane depolarization and apoptosis (P < 0.05, C vs. H_2_O_2_ and P < 0.05, H_2_O_2_ vs. DOX+ H_2_O_2_; Fig 2B).

### Doxycycline improves left ventricular function and moderates left ventricular hypertrophy in ISO treated rats

**Fig 3:**
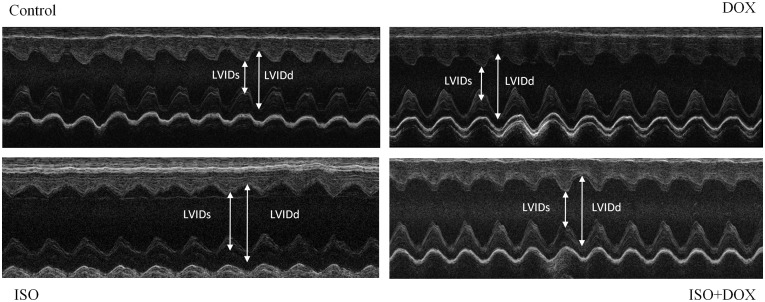
Representative echocardiographic M-mode images of left ventricles of control, DOX, ISO and ISO+DOX groups.

The echocardiographic parameters of animals did not differ significantly from each other at the beginning of the study. Heart rate did not differ significantly among the groups during the anesthesia. The thickness of the septum and posterior wall were also increased in the ISO group (indicating the presence of ventricular hypertrophy) compared to the control group (P < 0.05, ISO vs. C). Doxycycline treatment significantly reduced these unfavorable alterations. Systolic left ventricular function (EF %) and fractional shortening (FS %) were significantly lower in the ISO group (P < 0.05, ISO vs. C) and these deterioration was significantly improved by doxycycline administration (P < 0.05, ISO vs. ISO+DOX; Table 1; Fig 3).

**Table 1:** Effects of doxycycline on the echocardiographic parameters.

	Baseline	Control	DOX	ISO	ISO+DOX
**EF (%)**	76.42 ± 3.58	73.75 ± 1.39	72.81 ± 1.51	58.84 ± 1.27^#^	68.39 ± 0.94*
**FS (%)**	46.93 3.29	44.21 ± 1.41	43.62 ± 1.32	33.29 ± 0.19	39.96 ± 0.56*
**Septum (mm)**	1.52 ± 0.05	1.55 ± 0.04	1.46 ± 0.04	1.79 ± 0.08^#^	1.57 ± 0.04*
**PW (mm)**	1.50 ± 0.12	1.49 ± 0.74	1.42 ± 0.03	1.72 ± 0.05^#^	1.51 ± 0.04*
**LLVIDd (mm)**	8.21 0.21	8.64 ± 0.29	8.30 ± 0.18	8.46± 0.07	8.31 ± 0.22
**LVIDs (mm)**	4.36 ± 0.33	4.77 ± 0.09	4.69 ± 0.19	5.34 ± 0.07^#^	4.84 ± 0.12*
**LVEDV (ul)**	365.83 ± 20.43	415.11 ± 32.63	375.50 ±17.64	371.34 ± 8.83	387.42 ± 22.42
**LVESV (ul)**	86.31 ± 15.86	107.52 ± 5.73	103.24 ± 9.85	147.16 ± 3.26^#^	121.42 ± 4.86*
**LV mass (mg)**	991.69 ± 58.51	989.30 ± 63.91	953.00 ± 21.05	1212.24 ± 48.30^#^	990.93 ± 59.33*

Control group (C) (n = 5); doxycycline group (DOX) (n = 6); isoproterenol group (ISO) (n = 7); ISO+ doxycycline group (ISO+DOX) (n = 7). EF: ejection fraction, FS: fractional shortening, PW: posterior wall, LVIDd: diastolic left-ventricular inner diameter, LVIDs: systolic left-ventricular inner diameter, LVEDV: left ventricular end-diastolic volume, LVESV: left-ventricular end-systolic volume, LV mass: calculated left vantricular mass. Values are mean ± S.E.M.

^#^P < 0.05 vs. Control,

*P < 0.05 vs. ISO.

### Doxycycline treatment improves the gravimetric parameters in an ISO–induced heart failure model

There was no significant difference in body weight between the groups at the beginning or the end of the experiment. Gravimetric measurements were performed and significantly elevated ventricular weight (WV, g, C: 1.27 ± 0.02; DOX: 1.28 ± 0.02; ISO: 1.46 ± 0.03; ISO+DOX: 1.31 ± 0.02; P < 0.05, C vs. ISO) as well as ventricular weight normalized to body weight (WV/BW, mg/g, C: 2.28 ± 0.12; DOX: 2.27 ± 0.09; ISO: 2.87 ± 0.13; ISO+DOX: 2.39 ± 0.06; P < 0.05, C vs. ISO) and to tibia length (TL) (WV/TL, mg/mm, C: 25.54 ± 0.28; DOX: 26.51 ± 0.69; ISO: 29.95 ± 0.47; ISO+DOX: 26.66 ± 0.92; P < 0.05, C vs. ISO) were detected. Doxycycline treatment prevented the unfavorable changes in gravimetric parameters in the ISO+DOX group (P < 0.05, ISO+DOX vs. ISO) (Table 2).

**Table 2:** Effect of doxycycline on the Ventricular Weight/Tibia Length (VW/TL) ratio and on plasma BNP.

Group	Control	DOX	ISO	ISO+DOX
**Weight (g)**	564.20 ± 21.55	569.67 ± 23.04	515.71 ± 19.66	550.86 ± 14.89
**Ventricular weight (g)**	1.27 ± 0.02	1.28 ± 0.02	1.46 ± 0.03^#^	1.31 ± 0.02*
**Tibia length (mm)**	48.00 ± 0.55	48.50 ± 0.76	48.86 ± 0.51	49.43 ± 0.49
**Ventricular weight/body weight (mg/g)**	2.28 ± 0.12	2.27 ± 0.09	2.87 ± 0.13	2.39 ± 0.06*
**Ventricular weight/tibia length (mg/mm)**	25.54 ± 0.28	26.51 ± 0.69	29.95 ± 0.47^#^	26.6 ± 0.92*
**p-BNP (ng/ml)**	1.62 ± 0.11	1.61 ± 0.01	2.29 ± 0.06^#^	1.66 ± 0.10*

Eight weeks after ISO–induced myocardial infarction, body weight, mass of ventricles and tibia length were measured. Ventricular weight/body weight (mg/g) and ventricular weight/tibia length (mg/mm) ratios were calculated. Plasma B-type Natriuretic Pepetide (p-BNP) level was determined by ELISA. Control group (C) (n = 5); doxycycline group (DOX) (n = 6); isoproterenol group (ISO) (n = 7); ISO+ doxycycline group (ISO+DOX) (n = 7). The results were expressed as mean ± S.E.M.

^#^P < 0.05 vs. Control,

*P < 0.05 vs. ISO.

### Doxycycline inhibits the heart failure–induced elevation of plasma BNP level

Elevation in plasma BNP levels was strongly associated with the severity of heart failure. The BNP level increased significantly in the ISO group 8 weeks after myocardial infarction (P < 0.05, C vs. ISO). However, doxycycline could significantly reduce the plasma BNP level (P < 0.05, ISO vs. ISO+DOX) suggesting that doxycycline decreased the severity of postinfarction heart failure. There was no significant difference between the control and the DOX groups (Table 2).

### Doxycycline decreases the interstitial collagen deposition in the myocardium

**Fig 4:**
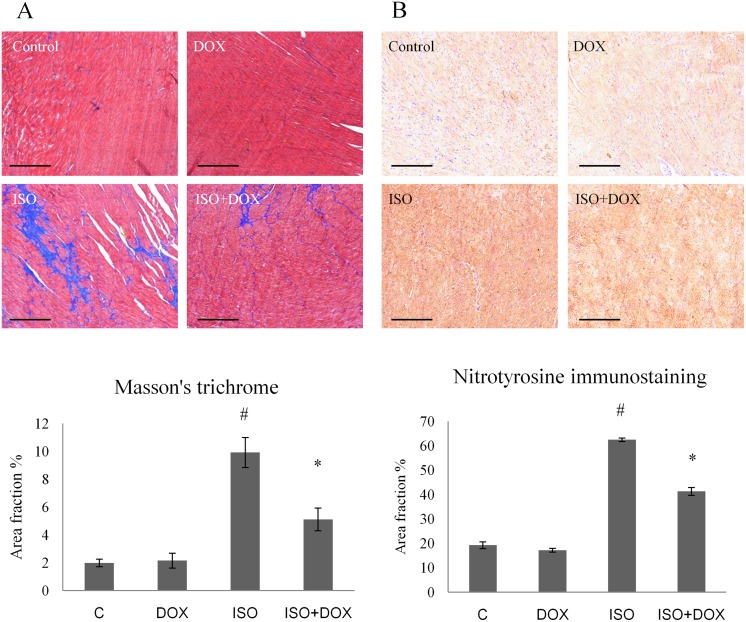
DOX reduces ISO–induced interstitial collagen deposition and protein nitrosylation in ISO–induced heart failure. **(A)** Sections stained with Masson’s trichrome (scale bar: 500μm, magnifications 5-fold). Control: age-matched rats, DOX: age-matched animals treated with doxycycline for 8 weeks, ISO: age-matched animals 8 weeks after ISO administration, ISO+DOX: age-matched animals treated with doxycycline, 8 weeks after ISO administration. Values are mean ± SEM. #P < 0.05 (ISO vs. control group), *P < 0.05 (ISO+DOX vs. ISO group). **(B)** Representative immunohistochemical stainings for nitrotyrosine (NT, brown staining, scale bar: 500μm, magnifications 5-fold) in the myocardium of control: age-matched rats, DOX: age-matched animals treated with doxycycline for 8 weeks, ISO: age-matched animals 8 weeks after ISO administration, ISO+DOX: age-matched animals treated with doxycycline, 8 weeks after ISO administration. Values are mean ± SEM. #P < 0.05 (ISO vs. control group), *P < 0.05 (ISO+DOX vs. ISO group).

Histological analysis revealed marked scar tissue formation after ISO stress in failing rat hearts compared to the control group (P < 0.05). Doxycycline treatment significantly decreased the extent of interstitial fibrosis (P < 0.05). Doxycycline alone did not cause any alterations in myocardial hypertrophy or interstitial collagen deposition (Fig 4A).

### Effects of doxycycline on the oxidative stress marker nitrotyrosine

The presence of oxidative stress was been confirmed in our rodent heart failure model by the investigation of a liquid peroxidation product, NT. Myocardial sections from the control group showed almost no immunostaining for NT. In contrast, in animals with heart failure (ISO), immunostaining notably increased (P < 0.05, C vs. ISO), but this increase was attenuated by DOX treatment (P < 0.05, ISO vs. ISO+DOX; Fig 4B).

### Effect of doxycycline on the expression of OPA-1 and Mfn-2 and phosphorylation of Drp-1^Ser616^

**Fig 5:**
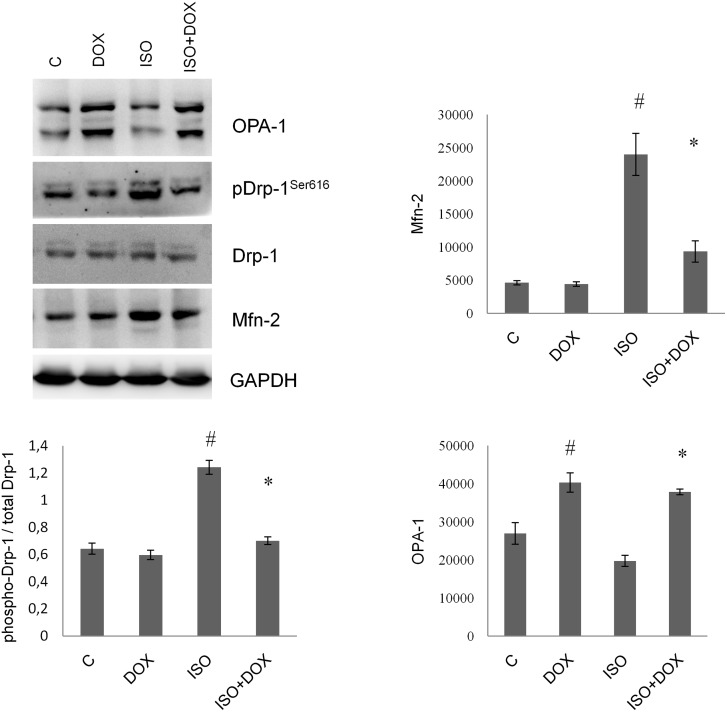
DOX favorably modulates the expression of Mfn-2, OPA-1 and the phophoryltaion of Drp-1. Representative western blot analysis of Mfn-2, OPA-1, Drp-1 and pDrp-1^Ser616^ and densitometric evaluation is shown. PhosphoDrp-1^Ser616^ bands were normalized to the appropriate Drp-1 bands. Representative blots and bar diagrams of three independent experiments are presented. C: control animals, ISO: animals 8 weeks after ISO administration; ISO + DOX: animals treated with doxycycline, 8 weeks after ISO administration; DOX: animals treated with doxycycline for 8 weeks. Values are mean ± SEM. # P < 0.05 vs control, *P < 0.05 vs. ISO.

OPA-1 expression significantly increased in the DOX treated groups compared to the control and ISO groups (P < 0.05, control vs. DOX and P < 0.05, ISO vs. ISO+DOX) whereas the expression of Mfn-2 decreased significantly in the ISO+DOX group compared to the ISO group (P < 0.05, ISO vs. ISO+DOX). The phosphorylation level of Drp-1^Ser616^ increased significantly in the ISO group and decreased significantly in the ISO+DOX group (P < 0.05, C vs. ISO and P < 0.05, ISO vs. ISO+DOX). Protein levels were measured with Nanodrop and GAPDH was used as loading control (Fig 5).

## Discussion

We investigated the effect of DOX on H9c2 cardiomyocyte cell culture and on an isoproterenol—induced heart failure animal model. Subcutaneous administration of isoproterenol produces diffuse myocardial necrosis predominantly subendocardially [31, 32]. The significant myocardial cell loss is followed by hypertrophy and remodeling leading to impaired left ventricular function and heart failure similar to that observed in post-MI patients. LV systolic function is a strong predictor of cardiac mortality and morbidity [33]. ISO treatment significantly decreased the systolic left ventricular function, elevated ventricular wall thickness and diameter and caused significant myocyte hypertrophy. DOX was capable of preserving LV systolic function (EF%, FS%) and normalized wall thickness and ventricular diameter (Table 1, Fig 3). In accordance with these results, plasma BNP levels increased in the ISO–treated animals, and this increase was attenuated by DOX treatment (Table 2). There are data in the literature about the protective effect of doxycycline on isoproterenol induced cardiac hypertrophy and on the early phase of remodeling [22, 23]. Our working group verified for the first time that DOX strikingly reduces the severity of postinfarction heart failure in an advanced stage.

Our histological examinations showed that ISO treatment induced excessive fibrosis, which was highly reduced by DOX treatment (Fig 4A). This effect is presumably based on its matrix metalloproteinase inhibition which has been described previously [34, 35]. The effect of DOX was so strong in our animal model, that we wanted to further clarify its action on myocardial protection. Recently emerging evidence indicates that myocardial oxidative stress contributes to heart failure [36, 37]. To establish the presence of oxidative damage in our animal model, we performed nitrotyrosine immunohistochemical analysis on the rat heart samples. Rats treated with ISO showed increased ROS production, which was highly attenuated by DOX treatment (Fig 4B).

There are some data in the literature about the antioxidant and scavenging properties of doxycycline [38]. Doxycycline has a multiple-substituted phenol ring which is the key to its ROS-scavenging abilities. The reaction of the phenol ring with a free radical generates a phenolic radical that becomes relatively stable and unreactive [39]. It is also supposed that doxycycline may inhibit MMPs by attenuating the oxidative stress [40].

Since mitochondria are a major source (and target) of ROS and play a critical role in energy production, we turned our attention to these organelles. Intensive research on the mitochondria has previously demonstrated that their structural and functional integrity is essential for maintaining normal myocardial function [15]. The tightly controlled balance between mitochondrial fusion and fission is important in high energy demanding cells, such as cardiomyocytes. Defects in mitochondrial dynamics have been associated with various disorders, including heart failure, ischemia/reperfusion injury, diabetes, and aging [7]. We first tested the effect of DOX on a H9c2 cardiomyocyte cell culture, where DOX protected cardiomyocytes against oxidative injury in the SRB assay (Fig 1). Next we examined the effect of DOX on ROS–induced mitochondrial fragmentation and membrane potential. Our results showed that DOX protected mitochondria against ROS–induced mitochondrial fragmentation (Fig 2A) and prevented ROS–induced collapse of the mitochondrial membrane potential (Fig 2B), processes that cause rapid impairment of mitochondrial and cellular function leading to necrotic and apoptotic cell death [41–43].

We further investigated the effect of DOX on the expression of dynamin-like GTPases (OPA-1, Mfn-2, DRP-1) responsible for mitochondrial dynamics in western blot analysis of rat heart samples. It has been reported that Mfn-2 is markedly induced by oxidative stress in H9c2 cardiomyocytes and Mfn-2 creates physical connection between mitochondria and endoplasmic reticulum [44]. After mitochondria is connected to the endoplasmic reticulum, a high amount of calcium enters the mitochondria from the ER and induces mitochondrial fragmentation [45, 46]. It is also known that elevated steady state level of OPA-1 leads to mitochondrial fusion and improves cell survival. On the contrary DRP-1 phosphorylation on Ser616 leads to mitochondrial fragmentation. It was reported earlier that pharmacological inhibition of Drp-1 attenuates mitochondrial membrane depolarization and protects the heart from ichemia/reperfusion injury, and Drp-1 expression is reduced in Mfn-2 knockout hearts [10, 11]. Reduced expression of Mfn-2 makes mitochondria more tolerant to oxidative stress and Ca^2+^ overload [44]. In our animal model, ISO treatment decreased OPA-1 and increased Mfn-2 levels, and augmented the phosphorylation of Drp-1. DOX was able to favorably modulate the steady state level of OPA-1, Mfn-2 and the phosphorylation of Drp-1, which prevented mitochondrial fragmentation and improved cell survival (Fig 5).

Some mitochondria targeted drugs such as PT pore inhibitors, NO analogs, antioxidants, potassium channel openers, metabolic modulators, have been examined in various clinical trials [47, 48]. Unfortunately, many of these drugs have failed, while others are still under investigation [49, 50], showing that effective modification of this fine balance in mitochondrial metabolism is difficult.

According to our results, DOX might be a promising agent in the treatment of postinfarction heart failure. Besides its well-known protective effect on cardiac hypertrophy, remodeling and fibrosis, we could demonstrate that DOX was able to decrease the ROS-induced mitochondrial fragmentation and depolarisation on H9C2 cardiomyocytes, and beneficially modulated the steady state level of OPA-1, Mfn-2 and the phosphorylation of Drp-1 in our postinfarction heart failure model. Further investigations are needed to better understand its protective effect on mitochondrial metabolism, ROS production and cell survival.
